# The Limits of the Dichotomy Between Responders and Nonresponders in Chronic Kidney Disease

**DOI:** 10.3390/jcm15208095

**Published:** 2026-10-19

**Authors:** Antonio Granata, Rosario Maccarrone, Alfio Edoardo Giuffrida, Leonardo Spatola, Francesco Pesce, Settimio Di Gregorio, Matthias Zeiler, Yuri Battaglia

**Affiliations:** 1 Nephrology and Dialysis Unit, “Cannizzaro” Emergency Hospital, 95126 Catania, Italy; antonio.granata4@tin.it (A.G.); rosario.maccarrone88@gmail.com (R.M.); alfiogiuffrida91@libero.it (A.E.G.); 2 Advanced School of Ultrasound in Nephrology, Italian Society for Ultrasound in Medicine and Biology (SIUMB), 00192 Catania, Italy; 3 Nephrology and Dialysis Unit, “St. Antonio Abate” Hospital, 91016 Trapani, Italy; leonardospatola9@gmail.com; 4 Nephrology, Dialysis and Transplantation Unit, Department of Emergency and Organ Transplantation, University of Bari “A. Moro”, 70121 Bari, Italy; francesco.pesce2@unicatt.it; 5 Department of Medicine, University of Verona, 37131 Verona, Italy; settimiodigregorio3@gmail.com; 6 Nephrology and Dialysis Unit, Department of Internal Medicine, “C. e G. Mazzoni” Hospital, 63100 Ascoli Piceno, Italy; matthias.zeiler@sanita.marche.it; 7 Department of Medicine and Health Sciences, University of Trento, 38122 Trento, Italy; 8 Nephrology and Dialysis Unit, Santa Chiara Hospital, ASUIT, 38122 Trento, Italy

**Keywords:** chronic kidney disease, treatment response, responder, nonresponder, albuminuria, eGFR, causal inference, treatment heterogeneity, surrogate endpoints, precision medicine

## Abstract

**Background/Objectives:** In contemporary chronic kidney disease care, determining whether an individual patient is benefiting from treatment is increasingly complex. Disease-modifying therapies may influence multiple clinical domains, and these effects may emerge with different magnitudes and time courses. Nevertheless, treatment response is frequently summarized using binary labels such as ‘responder’ and ‘nonresponder’, often on the basis of a single biomarker at a single time point. Such classifications are useful when associated with a clearly defined outcome, threshold, and assessment window, but may be misleading when interpreted as the patient’s overall response to therapy. **Methods:** This study examines the limitations of responder–nonresponder dichotomy, considering biological and analytical variability, treatment exposure, adherence, concomitant therapy, intercurrent events, and layered treatment. A domain-by-time framework for interpreting treatment-associated trajectories is proposed. **Results:** The framework first considers the nature and timing of observed changes, then assesses their interpretability considering treatment exposure and clinical context, and finally evaluates the strength of their attribution to treatment. It provides a conceptual, patient-level reporting and research framework that complements existing estimands principles, validated surrogate endpoints, and disease-specific response criteria. **Conclusions:** The proposed framework aims to improve the description and interpretation of treatment-associated trajectories without implying individual causal treatment effects. Its reproducibility and clinical utility require prospective evaluation.

## 1. Introduction

The availability of effective therapies for chronic kidney disease (CKD) has expanded substantially in recent years. The renin–angiotensin system inhibition model is now complemented by sodium-glucose cotransporter 2 inhibitors, non-steroidal mineralocorticoid receptor antagonists and, in appropriate populations, glucagon-like peptide-1 receptor agonists [1]. This progress has profoundly changed the management of CKD, but it has also made an apparently simple question increasingly difficult to answer. The question that arises is how to ascertain whether an individual patient is truly benefiting from treatment. Real-world observational studies have documented opportunities to improve the use of nephroprotective therapies and have evaluated the efficacy and safety of dapagliflozin in routine CKD care [2,3].

The issue pertains to the fact that modern therapeutic interventions capable of modifying disease progression and clinical outcomes do not act upon a single parameter. These factors may potentially impact albuminuria, kidney function, blood pressure, cardiovascular risk, metabolic variables, tolerability, and safety. The magnitude and time course of these effects may differ across domains, treatments, and individuals. The effects span several response domains, each representing a distinct biological or clinical dimension through which treatment response may be observed. Concurrently, there has been an increasing trend in the combination of various therapies or their sequential introduction in the therapeutic regimen. Consequently, treatment has become multidimensional and layered, whereas the response of an individual patient is frequently summarized by the change in a single biomarker measured at a single point in time. The availability of mechanism-specific agents, including anticomplement therapies for selected kidney diseases, further emphasizes the need to specify the treatment and disease context when interpreting response [4]. This tension is particularly evident in the language of responder and nonresponder. A patient whose urinary albumin-to-creatinine ratio (UACR) falls beyond a prespecified threshold may be classified as a responder, whereas another who does not reach that threshold may be considered a nonresponder. Such definitions are not inherently problematic. When linked to a clearly defined parameter, threshold, and assessment window, they can be useful for describing a pharmacodynamic response, applying a treatment algorithm, selecting a study population, or communicating a specific result. The issue arises when a response observed in one domain is permitted to represent the patient’s overall response to treatment. Illustrative clinical and methodological studies indicate why this simplification may be inadequate. Albuminuria and blood pressure responses to renin–angiotensin system blockade can diverge within the same individual [5], concordance across several treatment-modifiable risk markers is limited [6], and randomized crossover studies demonstrate that the same patient may show different albuminuria responses to different therapeutic classes [7]. Nephrology is already moving beyond the single-biomarker approach. Dynamic biomarkers, multimarker and longitudinal assessments, crossover studies, response-guided strategies and, where feasible, N-of-1 designs are being explored to move from population-average efficacy toward more individualized kidney medicine [8]. The potential prognostic and predictive roles of omics-based and cardiovascular biomarkers in CKD have also been examined in focused reviews [9,10]. Albuminuria and chronic estimated glomerular filtration rate (eGFR) slope have been evaluated jointly as surrogate endpoints [11], while an exploratory SONAR analysis used UACR response and subsequent eGFR trajectory to identify contrasting subgroups for proteomic investigation [12]. This exploratory sampling strategy does not establish the feasibility of routine clinical trajectory classification. While these advances are significant, they do not fully resolve the interpretive problem. The central question is not simply whether one biomarker or several should be used, but how a change observed in an individual patient should be interpreted. The interpretation of the results is dependent on the changes that have occurred, the timing of the assessment, the extent of treatment exposure, and the clinical factors that may have influenced the observed trajectory. The reliability of attributing this change to treatment also depends on the confidence levels that are attached to the findings. The present perspective is a selective, conceptual narrative synthesis. We propose a domain-by-time framework for interpreting treatment-associated trajectories. The framework first establishes what changed and when. It then considers whether the observed trajectory can be meaningfully interpreted in light of treatment exposure and clinical context. Finally, it asks how confidently the change can be attributed to treatment. Its proposed contribution is a patient-level descriptive and reporting sequence that integrates existing concepts into three interpretive steps: description (what changed and when), interpretability (whether baseline status, measurement quality, treatment exposure, and clinical context allow the trajectory to be meaningfully interpreted), and attribution (how strongly the observed change can be linked to treatment). It does not introduce a new causal estimand, an established response phenotype, or a validated treatment-selection rule. In this way, the framework distinguishes between what is observed and what can be inferred, from pharmacodynamic response and prognostic significance to treatment-effect heterogeneity and individual causal benefit. As CKD therapy becomes more multidimensional, layered and combined, greater precision in describing treatment response may provide an important foundation for truly individualized treatment strategies.

## 2. What Does a Responder Label Actually Tell Us?

Albuminuria provides perhaps the clearest example of why this distinction matters in practice. UACR is one of the most clinically valuable biomarkers in CKD and is one of the measures most frequently used to define treatment response. The evidence supporting this claim is substantial. A recent meta-analysis of individual-participant data, encompassing 48 randomised trials with 85,681 participants, has demonstrated that a 30% relative reduction in geometric mean UACR is associated with a 19% lower hazard of the clinical kidney endpoint [13]. In combination with analyses that consider albuminuria change and chronic eGFR slope, these findings support UACR as a reliable surrogate endpoint in suitable CKD trial settings [11,13]. The value of UACR is therefore not in question. It is vital to be precise in the interpretation of any change observed in a patient. Trial-level surrogate validity and patient-level treatment response address different questions. At the trial level, we are interested in determining whether interventions that produce larger average reductions in UACR also tend to produce greater effects on clinical kidney outcomes. At the individual level, however, the question is different. What conclusions can be drawn when a patient’s UACR falls by 30% or 40% after the commencement of treatment? At the very least, we can state that the UACR decreased during the period following treatment initiation. Depending on the study design and clinical context, this change may provide pharmacodynamic or prognostic information, but it does not establish the magnitude of causal kidney benefit experienced by the individual or indicate what occurred in other clinically relevant response domains. This distinction was already evident in IRMA-2, where changes in urinary albumin excretion and systolic blood pressure after irbesartan frequently diverged within the same patient [5]. Subsequent analyses expanded this observation to encompass albuminuria, blood pressure, potassium, haemoglobin, cholesterol, uric acid and other treatment-modifiable variables [6]. Consequently, a patient may satisfy a predetermined albuminuria-response criterion without demonstrating a concomitant change in blood pressure or another clinically significant parameter. The most accurate description is not that the patient responded globally, but rather that a response occurred within a defined domain. This remains the case even in situations where the biomarker in question has been robustly validated. An excellent surrogate endpoint does not necessarily provide a complete description of an individual patient’s overall treatment response. The same principle applies to eGFR, blood pressure, body weight, glycaemic measures, potassium, and patient-reported outcomes. Symptom burden and physical performance have been examined in kidney transplant recipients and people with Fabry disease, respectively, illustrating distinct patient-relevant assessments that cannot be inferred from a UACR change alone [14,15]. Each of these measures captures a clinically relevant but distinct dimension of response. The term *responder* is informative only if we have a clear understanding of what the *responder* refers to. Responder analyses also require care because dichotomizing a continuous measure can discard information and make results depend on the chosen cut-off [16]. An individual response label should therefore specify the measured domain, threshold, and time window rather than imply general or individual causal benefit (Table 1).

## 3. Observed Response and Treatment Effect Are Different Questions

The challenge of interpretation is further heightened when a change observed after the start of treatment is deemed to be a result of the therapy. If there is a 40% decrease in UACR after treatment has been initiated, its causal meaning may not be immediately apparent. It is not possible to ascertain how UACR would have evolved in the same patient, over the same period, had treatment not been given. This unobserved counterfactual is central to causal inference.

Furthermore, the observed difference before and after treatment does not necessarily reflect the pharmacological effect alone. Several factors may contribute to the observed trajectory, including biological variability, analytical error, regression to the mean, adherence, changes in concomitant therapy, dietary behaviour, volume status, blood pressure and intercurrent illness. Consequently, a change that occurs during treatment should not be automatically interpreted as an individual causal treatment effect.

Methodological work on individual treatment response has highlighted this distinction for decades [17,18]. The ICH E9(R1) estimand framework specifies population, treatment conditions, outcome variable, strategies for handling intercurrent events, and population-level summary measure [19] (Table 1). Its explicit attention to measurement timing and treatment changes parallel components of the proposed framework, but an estimand targets a defined comparative treatment effect rather than an observed individual trajectory. Similarly, the PATH statement provides methods for evaluating predictive heterogeneity of treatment effects from comparative trial data [20] (Table 1). The domain-by-time approach does not replace these methods; it instead structures patient-level observations before any prognostic or causal inference is attempted.

**Table 1:** Relationship of the proposed framework to established approaches (conceptual comparison).

Existing Approach	What It Addresses/Limitation	Proposed Patient-Level Contribution
ICH E9(R1) estimands [19]	Specify population-level comparative questions, including treatment conditions and intercurrent events; do not directly categorize a single observed patient trajectory.	Document observed domains, relative timing and exposure context before considering causal attribution.
Responder analyses [16]	Summarize the proportion crossing a specified threshold; a binary split can obscure continuous change and variability.	Require the domain, cut-off, assessment window and measurement uncertainty alongside any responder label.
Predictive HTE methods (PATH) [20]	Estimate differential comparative treatment benefit using trial-based risk/effect models; individual before–after change is insufficient.	Avoid interpreting descriptive cross-domain patterns as evidence of differential causal benefit.
Longitudinal markers and validated surrogates [11,13]	Relate trial-level intermediate outcomes to clinical endpoints; do not establish the causal effect in an individual.	Record potentially discordant domains and time courses without replacing validated endpoints.

Conventional parallel-group trials are well suited to estimating average treatment effects, but they provide limited information about whether a response observed in one patient is stable and reproducible within that individual. When treatment effects are reversible, outcomes change sufficiently rapidly, and the underlying condition remains reasonably stable, replicated crossover designs can provide stronger evidence. CKD makes this problem particularly tangible because commonly used biomarkers differ substantially in short-term biological variability. In clinically stable patients with CKD, the within-person coefficient of variation has been estimated at approximately 32.5% for first-morning UACR, compared with 5.4% for serum creatinine and 4.1% for cystatin C [21]. A patient whose UACR is close to a binary response threshold may therefore move from one category to another on repeat testing without a comparable change in the underlying biological process. Although thresholds can still offer clinical benefits, they do not guarantee precise measurements due to inherent variability. In the same study, the negative reference change value for first-morning UACR was approximately −58%, illustrating that a single 30% reduction may be within short-term measurement variability [21]. Where feasible, standardized collection and repeated baseline and follow-up samples should be used, and observed change should be interpreted in relation to the assay and within-person variability rather than a threshold alone. These variability estimates do not define a universal clinical-response cut-off. Evidence of within-person repeatability also exists: in an analysis involving repeated exposure to the same drug and dose, albuminuria responses correlated on re-exposure (R^2^ = 53% for lisinopril and 63% for losartan) [22]. These findings support study of within-person consistency but do not establish that a particular response profile predicts a larger causal benefit.

SONAR offers a valuable clinical illustration. During the active run-in phase, patients were classified according to their early UACR reduction with atrasentan. Early albuminuria change provided prognostic information, but it did not clearly identify patients who derived greater kidney benefit from randomized treatment [23]. More recent exploratory work from SONAR added a longitudinal dimension by combining short-term UACR response with subsequent eGFR trajectory [12]. This exploratory analysis selected contrasting UACR/eGFR subgroups for proteomic comparisons; it does not demonstrate routine clinical feasibility or validated trajectory-based treatment decisions. It nonetheless underscores a fundamental distinction. A category based on an observed change is not necessarily a category of differential causal treatment benefit.

For this reason, we use observed treatment-associated trajectory as the default descriptive term. It describes what happened after treatment initiation without assuming that the observed change was caused entirely by treatment. From that starting point, stronger conclusions require stronger evidence. In practical terms, confidence in attribution increases when temporal compatibility and a plausible pharmacodynamic link are supported by adequate treatment exposure, repeated measurements, within-person reproducibility, and, when feasible, randomized or replicated crossover evidence. An observed biomarker change is a longitudinal measurement; a pharmacodynamic response additionally implies a plausible relationship to drug exposure, without establishing long-term clinical benefit. Prognostic information links an observed change to subsequent risk, whereas a predictive biomarker requires evidence that the comparative treatment effect differs according to that marker. Heterogeneity of treatment effect refers to variation in causal treatment effects, not simply to variation in observed before-and-after changes. A marker associated with future risk is prognostic, but is not necessarily predictive of differential comparative treatment benefit. The latter requires evidence from appropriate treatment comparisons, with treatment-by-marker interactions interpreted in the context of prespecification and uncertainty [20]. The individual causal treatment effect represents the strongest level of inference and should therefore be invoked with corresponding caution.

These distinctions are not merely semantic. They define what can reasonably be concluded from an observed treatment-associated change and provide the terminology used throughout this Perspective (Table 2).

## 4. Response Has Both a Domain and a Time Dimension

**Figure 1:**
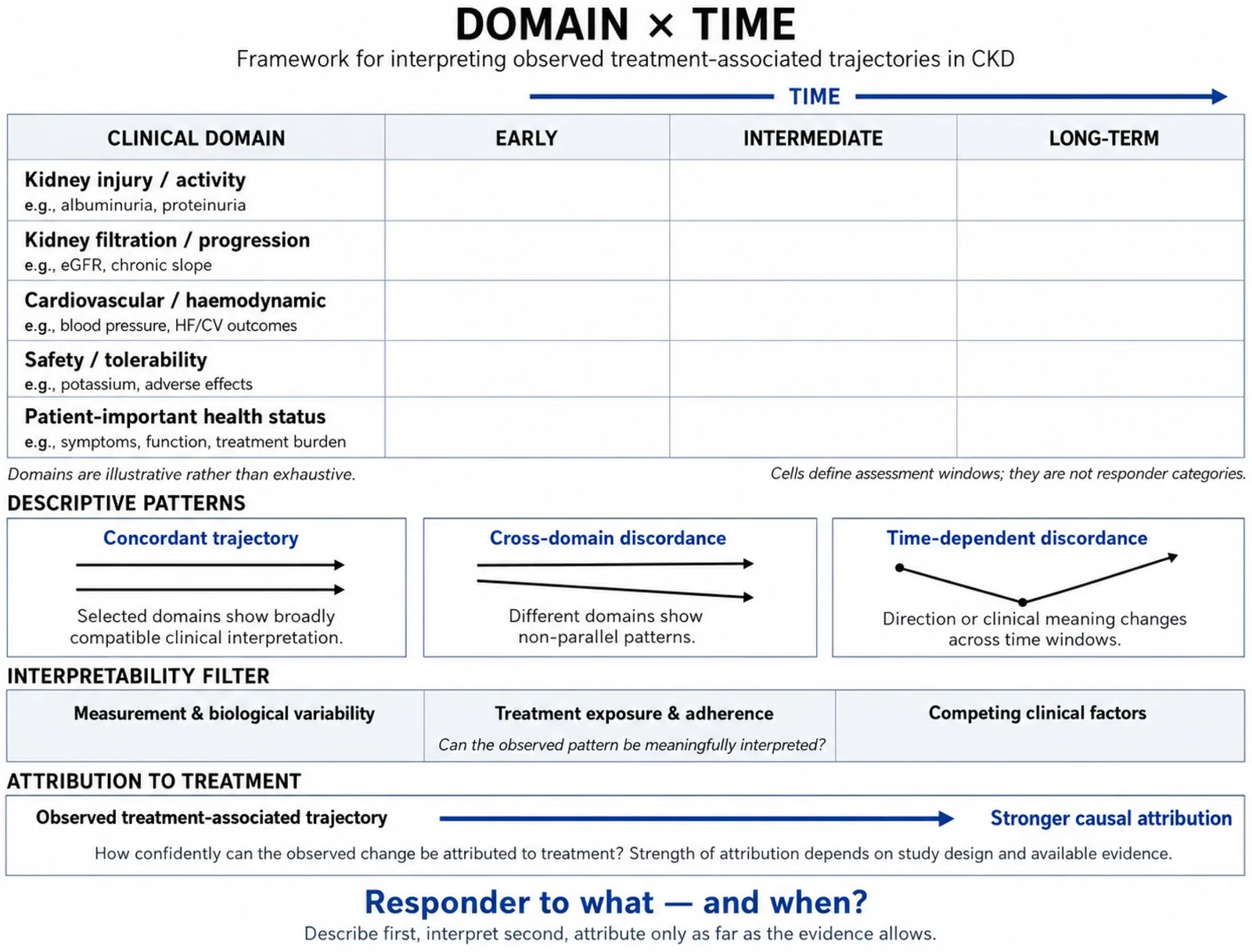
Domain-by-time framework for interpreting treatment-associated trajectories in CKD. Early, intermediate, and long-term are relative, treatment-marker-specific assessment windows, not fixed calendar intervals. Matrix cells indicate where observations would be placed. Table 3 provides a non-numeric, illustrative worked application. The trajectory labels are descriptive, not validated response phenotypes.

Once response is no longer regarded as a single, global property of the patient, its multidimensional nature becomes more apparent. Modern CKD therapies may influence albuminuria, kidney function, blood pressure, cardiovascular risk, metabolic variables, tolerability, and safety. However, these effects may not always evolve in parallel. These effects span distinct response domains, each capturing a specific biological or clinical dimension of treatment response. A favorable kidney biomarker trajectory may occur in conjunction with poor tolerability, and the absence of symptomatic improvement does not preclude a biologically meaningful kidney effect. The objective is not to transform these domains into a mandatory checklist or a universal score. In advanced CKD and dialysis, functional domains are also addressed in work on sarcopenia assessment, CKD exercise consensus recommendations, and randomized evidence on home-based exercise [24,25,26]. The relevance of these findings depends on the disease, intervention, population, and clinical question being investigated. It is essential to note that the domain must be specified prior to interpreting a response label. Finerenone exemplifies this distinction. In a mediation analysis of FIDELIO-DKD and FIGARO-DKD, a UACR reduction of at least 30% occurred in 53.2% of finerenone-treated participants and 27.0% of placebo-treated participants. Continuous UACR change was estimated to mediate 84% and 37% of the treatment effects on kidney and cardiovascular outcomes, respectively; when UACR change was dichotomized at 30%, these estimates decreased to 64% and 26%, respectively [27]. While these estimates do not establish individual-level discordance, they demonstrate that the same biomarker may capture different proportions of treatment effect depending on the outcome considered. Time introduces a second dimension. Treatment-associated changes may occur over days, months, or years, and the same variable may have a different clinical meaning depending on when it is measured. SGLT2 inhibition provides the clearest example of this phenomenon. In the prespecified EMPA-KIDNEY analysis, empagliflozin produced an acute eGFR reduction of approximately 2.12 mL/min/1.73 m^2^, corresponding to a dip of about 6% during the first two months, after which the chronic rate of eGFR decline was approximately halved [28]. Early UACR change may serve as a shorter-term pharmacodynamic readout, as illustrated in SONAR [23], while kidney or cardiovascular outcomes require substantially longer follow-up. Assessment windows should therefore be prespecified according to mechanism, marker, and clinical purpose. For interventions with delayed biological effects, the early window should not be defined solely by a conventional calendar cut-off; it should reflect known pharmacology, expected marker kinetics, and the relevant clinical endpoint. Distinct domains can have overlapping or nonmatching windows, and missing observations must not be interpreted as unchanged response. For example, the 6-week active run-in in SONAR represents an early UACR assessment window, whereas the first two months of EMPA-KIDNEY capture the acute eGFR phase rather than the chronic filtration trajectory [23,28]. The time axis can be kept simple (early, intermediate, and long-term) as long as these are understood as relative rather than fixed intervals. In practical terms, response is meaningful only when we know both what changed and when it was assessed. This is illustrated in Figure 1 by the question, “Responder to what and when?”

**Table 3:** Qualitative worked application of the domain-by-time matrix.

Domain/Starting Point	Early Window (Prespecified)	Intermediate Window	Longer-Term Assessment/Interpretation
Albuminuria: baseline UACR, repeated where feasible	Measure UACR using a comparable sample; do not interpret one threshold crossing alone.	Repeat UACR and record adherence and added or titrated therapies.	Assess whether change is sustained; a falling UACR is not an individual causal benefit estimate.
Filtration: baseline eGFR and prior trajectory	Measure eGFR for an acute dip after SGLT2 inhibitor initiation.	Reassess eGFR once the acute phase has passed.	Estimate chronic slope only with sufficient follow-up; distinguish early dip from subsequent progression.
Haemodynamic: baseline blood pressure	Document blood pressure and clinical volume status.	Review serial blood pressure, medication changes, and symptoms.	Interpret separately from UACR; a stable BP does not negate a kidney biomarker change.
Safety: baseline potassium and tolerance	Measure potassium and symptoms as clinically indicated after treatment initiation.	Review hyperkalaemia, adverse effects, treatment changes, and persistence.	Use established safety monitoring and treatment guidance, not unvalidated trajectory labels.
Exposure/context: prior therapies, disease severity and baseline variation	Record start dates, doses, medication adherence, illness and concurrent agents.	Reassess interruptions, titration, intercurrent events and assay comparability.	Attribute changes only to the extent supported by design; simultaneous drug initiation prevents separating component effects.

## 5. When Discordance Is Real and When It Is Apparent

Cross-domain and cross-time comparisons can make discordance easier to recognize, but not necessarily easier to interpret. Discrepancies may indicate authentic biological variations. The dissociation between albuminuria and blood pressure after renin–angiotensin system blockade is a well-established example [5,6], and randomized crossover studies show that the treatment producing the greatest albuminuria reduction may differ among individuals [7]. The trajectory of a treatment response is influenced by multiple factors, including the patient’s characteristics, the intervention’s specifics, and the domain in which the response is evaluated. However, discordance may also be apparent rather than biologically stable. It is important to consider measurement error and biological variability as potential sources of uncertainty, particularly for albuminuria, where values close to a response threshold may shift classification when measurements are repeated [21]. Regression to the mean may also be a contributing factor when treatment is initiated on the basis of an unusually high baseline value. The patient’s starting point matters: baseline UACR and eGFR severity, pretreatment variation, proximity to any response threshold, CKD etiology, prior therapy, and concurrent medication can alter the interpretation of the same absolute or relative change. For example, observational findings concerning dapagliflozin in Fabry-associated CKD should be interpreted in their disease-specific context rather than automatically generalized to other CKD populations [29]. Treatment exposure is equally important. Before concluding that a therapy has failed, it is necessary to determine if the intended dose was actually taken, for how long, with what degree of adherence and persistence, and whether adverse effects led to dose reduction or discontinuation. The clinical context may further influence the observed trajectory. Changes in diuretic or renin–angiotensin system therapy, glycaemic control, sodium intake, congestion, volume status, infection, or acute kidney injury may influence one domain more than another. Blood-pressure-directed interventions, such as renal denervation discussed in a CKD-specific position paper, further illustrate why the therapeutic target and the response domain must both be identified [30]. The presence of concomitant disease-modifying therapies can further complicate the attribution process. These are not peripheral details to consider after response has been classified. They determine whether the observed pattern is interpretable in the first place. For this reason, the term “nonresponder” should be used judiciously. Before attributing this label, it is imperative to first assess various domains, consider the temporal aspect, evaluate the patient’s treatment exposure, and explore plausible alternative explanations (Table 4).

These questions are deliberately simple. They are not intended to replace validated disease-specific definitions of response or remission, which may already integrate proteinuria, kidney function, concomitant treatment, and rescue therapy. Disease-specific instruments, such as the FAbry STabilization indEX, also illustrate why the purpose and evidentiary basis of a monitoring measure should be distinguished from a universal treatment-response label [31]. The framework addresses a more specific problem: the use of an unqualified responder label to summarize a treatment-associated change that has not been adequately specified in terms of domain, timing, exposure, and clinical context. For this reason, response pattern or treatment-associated trajectory is preferred. The terms concordant, cross-domain discordant, and time-dependent discordant are descriptive aids, not validated clinical categories or biological phenotypes. The phrase response phenotype would require separate evidence of within-person reproducibility and biological validity.

## 6. A Framework for Interpreting Treatment-Associated Trajectories

The preceding considerations can now be integrated into a unified framework without the need for an additional classification system. An observed treatment-associated trajectory is located at the intersection of a clinically relevant domain, such as kidney injury or activity, filtration and progression, cardiovascular or hemodynamic status, safety and tolerability, or patient-important health status, and a time window appropriate for the biomarker, intervention, disease mechanism, and clinical purpose. The domains shown are illustrative, not exhaustive. The Early, Intermediate, and Long-term axis is deliberately relative, reflecting the fact that the meaning of an observation may change across treatment phases rather than according to universal time cut-offs. Accordingly, these windows should be prespecified for each treatment-marker pair rather than treated as universal cut-offs. The matrix does not contain a responder category; rather, it contains the trajectory itself. It is deliberately unpopulated because assessment windows and domains must be selected for the treatment and clinical question; the accompanying worked example in Table 3 shows how individual cells can be used without asserting a validated response classification. Some trajectories may maintain their clinical coherence across the domains and time windows under consideration, while others may vary across domains or undergo semantic shifts over time. We describe these patterns as concordant, cross-domain discordant, and time-dependent discordant trajectories. Concordance refers to a compatible clinical interpretation rather than to identical numerical direction or magnitude. For example, a reduction in UACR without a concomitant blood-pressure change represents cross-domain discordance; an early eGFR decline followed by a slower chronic decline under SGLT2 inhibition illustrates time-dependent discordance; and a favorable kidney biomarker trajectory accompanied by poor tolerability reflects discordance between efficacy and safety domains [5,28]. These pattern descriptions are provisional, are not mutually exclusive, and are neither validated prognostic categories nor causal phenotypes. The interpretation of such findings remains contingent upon factors such as measurement and biological variability, treatment exposure, adherence to the prescribed regimen, and the impact of competing clinical factors. Only through a comprehensive and nuanced evaluation can we ascertain the biological significance of any apparent patterns. The framework also differs from a multimarker surrogate strategy. A joint assessment of albuminuria and chronic eGFR slope is necessary to determine whether intermediate randomized treatment effects improve the inference about clinical outcomes at the trial level [11]. Here the question is different and deliberately centered on the patient. When multiple changes are observed within the same individual, how should they be described without conflating pharmacodynamic response, prognosis, surrogate validity, treatment-effect heterogeneity, and individual causal benefit? This distinction becomes increasingly important as CKD therapy becomes more complex. Attribution is more challenging when multiple disease-modifying treatments are initiated, titrated, or discontinued across overlapping time windows. The CONFIDENCE trial demonstrated that the combination of finerenone and empagliflozin resulted in a more significant reduction in UACR compared to the individual treatments in patients with CKD and type 2 diabetes who were receiving renin–angiotensin system inhibition [32]. If two therapies are initiated simultaneously and UACR subsequently declines, the albuminuria trajectory can be characterized as occurring under the combination regimen. However, attributing that change to the regimen, or to a specific component, requires further evidence. It is important to note that response to the regimen is not synonymous with response to one component of the regimen. As combination therapy becomes more prevalent, it is essential to explicitly define the unit of inference.

An illustrative worked application is presented (no patient-level data or invented treatment effects). Consider a patient with albuminuric CKD and type 2 diabetes receiving background care and starting an SGLT2 inhibitor with finerenone. The initial status, baseline variation, dose and adherence, and the timing of each drug should be recorded. Table 3 translates the domains of Figure 1 into observations to collect alternative explanations and possible interpretations. The scenario is conditional and does not imply that the described trajectories necessarily occur in the same patient or justify changing therapy. For example, if repeated UACR measurements fall while potassium becomes elevated, the observations are descriptively cross-domain discordant; any safety action must follow established clinical guidance rather than an unvalidated response label. Thus, this exercise illustrates documentation and interpretation, not a proven improvement in treatment selection.

This issue is already entering clinical research. A small, decentralized feasibility study published in 2026 used dynamic UACR changes to guide the continuation of empagliflozin, the addition of finerenone, or the substitution of finerenone in patients with type 2 diabetes and CKD [33]. This feasibility study illustrates an investigational albuminuria-guided approach, but does not establish improvement in longer-term clinical outcomes; a UACR-guided strategy is, by definition, guided by albuminuria response. The reproducibility of this response, the identification of differential causal treatment benefit, and the improvement of long-term outcomes when used to guide treatment are separate questions that require prospective testing. Most examples discussed in this Perspective are derived from albuminuric CKD, particularly diabetic kidney disease, as these settings currently provide the most robust trial evidence. Applicability to nonalbuminuric CKD and other disease-specific settings should be tested rather than inferred from UACR-centered examples (Figure 1). In such settings, the relevant domains and assessment windows should be anchored to established disease-specific markers and validated response or remission criteria.

## 7. From Better Description to Testable Science

A conceptual framework becomes scientifically useful when it yields testable questions and specifies circumstances in which its assumptions would not be supported. The initial step is to ascertain whether apparent multidomain and time-dependent response patterns persist once biological variability and measurement error are mitigated. Prospective studies should therefore rely on repeated, standardized measurements and should prospectively characterize treatment exposure, adherence, persistence, concomitant therapies, and relevant clinical events. The initial objective is not to define new responder phenotypes, but rather to establish whether a reproducible signal remains after noise has been minimized. This requirement renders the framework falsifiable. If apparent cross-domain or time-dependent patterns largely disappear after repeated measurement and adequate characterization of treatment exposure, their biological relevance would be weakened. Similarly, if domain-by-time information adds no clinically meaningful information beyond established markers such as UACR and eGFR, its proposed incremental value would not be supported. A second question is whether an observed pattern is consistent within the same individual. When treatment effects are sufficiently rapid and reversible, replicated crossover designs may help determine whether the same treatment-associated pattern recurs after re-exposure. Randomized crossover comparisons and re-exposure studies in CKD provide relevant evidence about between-treatment differences and within-person response consistency [7,22]. However, consistency alone is not sufficient. Domain-by-time information must also provide clinically relevant predictive or treatment-selection information beyond that already available from established markers. The ultimate evaluation of this descriptive framework must address its clinical utility. Studies of lung-ultrasound-guided management in hemodialysis, including a randomized strategy and longer-term clinical outcome assessment, illustrate why the clinical value of a biomarker-guided care pathway requires explicit outcome evaluation [34,35]. A response pattern may be reproducible, biologically meaningful, and prognostically informative without improving treatment selection. Patient preferences and shared decision-making also warrant consideration when defining clinical utility, particularly in advanced kidney disease [36]. Prospective studies will therefore need to determine whether using dynamic response to guide therapy improves treatment choice, persistence, safety, or clinical outcomes compared with contemporary guideline-based care. A multicenter randomized trial of exercise in dialysis provides a further example of assessing a functional intervention through comparative evidence without equating that functional outcome with individual kidney protection [37]. The progression from description to possible response-guided care can thus be organized into four empirical tests: reproducibility, within-person consistency, incremental information, and clinical utility (Table 5). These tests form a logical evidentiary sequence, although individual components can be investigated in parallel. No universal thresholds for successful performance are proposed; clinically meaningful targets, comparators, and time horizons must be prespecified.

If the framework is unsuccessful at the earlier stages, it will be limited to a descriptive role. A more precise response description does not itself justify treatment modification. The use of this framework to guide treatment would only be justified by evidence that response-guided care improves clinically relevant outcomes compared with contemporary guideline-based care.

## 8. Conclusions

The responder–nonresponder distinction remains useful when linked to a defined domain, threshold, and assessment window, but it should not represent a patient’s overall treatment response. In CKD, treatment-associated trajectories may differ across clinical domains and time points and are influenced by biological variability, treatment exposure, concomitant therapies, and intercurrent events.

The proposed domain-by-time framework aims to improve the description and interpretation of these trajectories without implying individual causal treatment effects or replacing established response criteria. Its potential to improve treatment selection and patient outcomes remains unproven and requires prospective evaluation of reproducibility, within-person consistency, incremental information, and clinical utility. Until then, the framework should complement established CKD management rather than guide treatment decisions independently.

## Figures and Tables

**Table 2:** Terminology for treatment-associated change in CKD.

Term	Appropriate Meaning	What It Does Not Establish
Observed treatment-associated trajectory	Longitudinal change observed after treatment initiation or modification	That treatment caused the entire observed change in the individual
Pharmacodynamic response	Change in a biological readout plausibly linked to pharmacological exposure	Long-term clinical benefit
Prognostic information	Association between an observed change and subsequent outcome risk	That modifying the marker necessarily causes the outcome change
Surrogate endpoint	Intermediate endpoint validated to support inference about a randomized treatment effect on a clinical endpoint in a defined context	Magnitude of causal benefit experienced by an individual
Predictive biomarker	Baseline or dynamic characteristic associated with differential treatment effect, ideally supported by a treatment-by-marker interaction in randomized data	Simple association with prognosis
Heterogeneity of treatment effect	Variation in causal treatment effect across individuals or clinically defined strata	Dispersion of observed before-and-after changes alone
Individual causal treatment effect	Difference between the potential outcomes of the same individual under alternative treatment conditions	Direct observability in a conventional parallel-group trial

**Table 4:** Minimum information required before labeling nonresponse in CKD.

Baseline context	What are baseline severity, pretreatment variability, disease etiology, prior treatment, concurrent medicines, and proximity to the chosen threshold?
Measurement quality	Were UACR/eGFR and other measures collected comparably and repeated as needed to assess biological and analytic variability?
Domain	Which domain has failed to change, and what response definition is being applied?
Timing	Was the assessment performed within an appropriate window for the marker-treatment pair?
Exposure	Was adequate treatment exposure achieved, with sufficient adherence and persistence to make nonresponse interpretable?
Alternative explanations	Could measurement or biological variability, concomitant therapy, or competing clinical events explain the observed trajectory?

**Table 5:** Four empirical tests of a response framework in CKD.

Test	Question
Reproducibility	Do apparent response patterns persist after repeated, standardized measurements? Candidate analyses include within-person coefficients of variation, reference change values, intraclass correlation, and repeated-measures agreement; methods and acceptable agreement should be prespecified.
Within-person consistency	When re-exposure is feasible and ethically appropriate, does the pattern recur under the same regimen and assessment window? Replicated crossover or re-exposure studies may assess correlations and within-person agreement; any minimum consistency criterion must be justified in advance.
Incremental information	Does domain-by-time information add value beyond baseline risk factors, UACR, and eGFR? Compare prespecified models using external or internal-external validation, calibration and discrimination; assess treatment-by-pattern interaction in appropriate comparative data before making claims about differential benefit.
Clinical utility	In prospective comparative studies, does a domain-by-time-guided strategy improve clinically important outcomes versus guideline-based care? Prespecify decisions, harms, patient-reported outcomes, kidney outcomes and follow-up duration; decision-curve analysis alone cannot demonstrate clinical benefit.

## Data Availability

No new data were created or analyzed in this study. Data sharing is not applicable to this article.
